# Influence of human peripheral blood samples preprocessing on the quality of Hi-C libraries

**DOI:** 10.18699/VJGB-23-11

**Published:** 2023-03

**Authors:** M.M. Gridina, E. Vesna, M.E. Minzhenkova, N.V. Shilova, O.P. Ryzhkova, L.P. Nazarenko, E.O. Belyaeva, I.N. Lebedev, V.S. Fishman

**Affiliations:** Institute of Cytology and Genetics of the Siberian Branch of the Russian Academy of Sciences, Novosibirsk, Russia; Institute of Cytology and Genetics of the Siberian Branch of the Russian Academy of Sciences, Novosibirsk, Russia; Research Centre for Medical Genetics, Moscow, Russia; Research Centre for Medical Genetics, Moscow, Russia; Research Centre for Medical Genetics, Moscow, Russia; Tomsk National Research Medical Center of the Russian Academy of Sciences, Tomsk, Russia; Tomsk National Research Medical Center of the Russian Academy of Sciences, Tomsk, Russia; Tomsk National Research Medical Center of the Russian Academy of Sciences, Tomsk, Russia; Institute of Cytology and Genetics of the Siberian Branch of the Russian Academy of Sciences, Novosibirsk, Russia

**Keywords:** Hi-C, human peripheral blood, blood samples storage, Hi-C., периферическая кровь человека, хранение образцов крови

## Abstract

The genome-wide variant of the chromatin conformation capture technique (Hi-C) is a powerful tool for revealing patterns of genome spatial organization, as well as for understanding the effects of their disturbance on disease development. In addition, Hi-C can be used to detect chromosomal rearrangements, including balanced translocations and inversions. The use of the Hi-C method for the detection of chromosomal rearrangements is becoming more widespread. Modern high-throughput methods of genome analysis can effectively reveal point mutations and unbalanced chromosomal rearrangements. However, their sensitivity for determining translocations and inversions remains rather low. The storage of whole blood samples can affect the amount and integrity of genomic DNA, and it can distort the results of subsequent analyses if the storage was not under proper conditions. The Hi-C method is extremely demanding on the input material. The necessary condition for successfully applying Hi-C and obtaining high-quality data is the preservation of the spatial chromatin organization within the nucleus. The purpose of this study was to determine the optimal storage conditions of blood samples for subsequent Hi-C analysis. We selected 10 different conditions for blood storage and sample processing. For each condition, we prepared and sequenced Hi-C libraries. The quality of the obtained data was compared. As a result of the work, we formulated the requirements for the storage and processing of samples to obtain high-quality Hi-C data. We have established the minimum volume of blood sufficient for conducting Hi-C analysis. In addition, we have identified the most suitable methods for isolation of peripheral blood mononuclear cells and their long-term storage. The main requirement we have formulated is not to freeze whole blood.

## Abstract (ru)

Метод захвата конформации хроматина в его полногеномном варианте (Hi-C) – мощный инструмент не только для выявления закономерностей пространственной организации генома, но и для понимания влияния их нарушения на развитие заболеваний. Кроме того, метод может быть использован для детекции хромосомных перестроек, в том числе сбалансированных транслокаций и инверсий. Применение метода Hi-C для поиска хромосомных перестроек получает все более широкое распространение. Это связано с тем, что современные высокопроизводительные методы анализа генома позволяют эффективно детектировать точечные мутации и несбалансированные хромосомные перестройки. Однако чувствительность этих методов для определения сбалансированных транслокаций и инверсий остается достаточно низкой. Хранение образцов цельной крови может влиять на количество и целостность выделяемой из них геномной ДНК, а кроме того, приводить к искажению результатов последующих анализов в том случае, если хранение осуществлялось в ненадлежащих условиях. Метод Hi-C крайне требователен к исходному материалу, так как необходимым условием для его успешного применения и получения качественных данных является сохранение пространственной укладки хроматина внутри ядра. Цель нашего исследования состояла в том, чтобы определить оптимальные условия хранения крови для проведения последующего анализа Hi-C. Были выбраны 10 различных условий хранения образцов крови и пробоподготовки. Для каждого условия приготовлены Hi-C библиотеки и отсеквенированы, после чего оценивалось качество полученных библиотек. В результате сформулированы требования к хра- нению и подготовке образцов, необходимые для получения качественных Hi-C данных. Нами установлен ми- нимальный объем образца крови, достаточный для проведения Hi-C анализа. Помимо этого, мы определили способы выделения ядерных элементов крови и их долгосрочного хранения, наиболее подходящие для после- дующего проведения Hi-C анализа. Основное требование, сформулированное нами, – не замораживать цель- ную кровь

## Introduction

The combination of chromatin conformation capture methods with whole genome sequencing led to the development of a simple and efficient Hi-C protocol that allows genome-wide studying of chromatin architecture (Lieberman-Aiden et al., 2009; Rao et al., 2014). In addition to the data concerning the organization and dynamics of chromatin in the nucleus, the Hi-C results showed that the relationship between three-dimensional distance in nuclear space and “nucleotide” distance in genomic coordinates can be described by a power function for all cell types. This means that chromosomal rearrangements have effects not only on the contacts frequency of regions directly located at the points of chromosome breaks, but also change the pattern of three-dimensional contacts of a wide area around the rearrangement boundary (Mozheiko, Fishman, 2019). Chromosomal rearrangements detecting methods based on the analysis of the chromatine three-dimensional organization have recently been proposed (Harewood et al., 2017; Chakraborty, Ay, 2018; Díaz et al., 2018; Fishman et al., 2018; Melo et al., 2020). These methods detect various types of rearrangements, including balanced ones, which are still difficult to detect by other methods (Hakim et al., 2012; Dong et al., 2017). In addition, information about single nucleotide variations can be obtained from Hi-C data (Mozheiko, Fishman, 2019), which is important for medical genetics

Whole blood is a common biological starting sample for medical genetics. Proper blood samples handling is critical for genome-wide studies. Long-term storage and inadequate storage conditions lead to a decrease in the amount of isolated DNA (Nederhand et al., 2003; Malentacchi et al., 2015; Schröder, Steimer, 2018) and its degradation (Ross et al., 1990; Permenter et al., 2015). A high degree of DNA degradation is a serious problem for subsequent molecular biological analyses (Palmirotta et al., 2011; Malentacchi et al., 2015). For example, an increase in the storage time of a blood sample leads to an overestimation of the level of DNA methylation, which may be due to the different stability of methylated and unmethylated DNA (Schröder, Steimer, 2018).

The key steps of the Hi-C protocol are chromatin fragmentation and ligation. To obtain high-quality datasets, it is necessary that both of these steps take place in nucleus, under conditions of maximum preservation of the nucleus integrity. Thus, unlike DNA sequence analysis methods, the Hi-C method imposes additional requirements on the quality of the input material. In this regard, it seems relevant to determine the appropriate storage conditions for blood samples intended for Hi-C analysis.

## Materials and methods

**Fig. 1.**
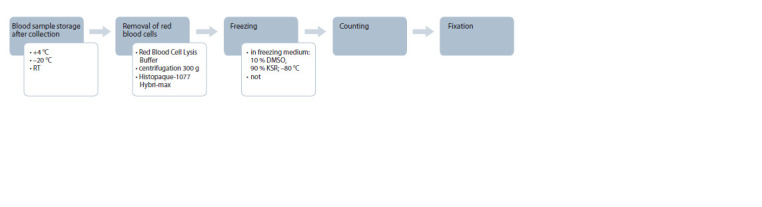
Blood samples preprocessing

Peripheral human blood was collected from the antecubital vein into Vacutainer EDTA Blood Collection Tubes. Blood samples storage conditions and preprocessing steps are specified in the Table and Figure 1.

**Table 1.**
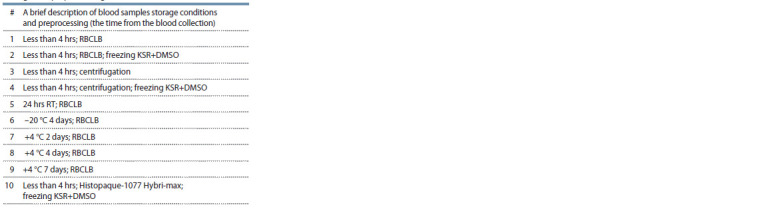
Storage and preprocessing conditions

The isolation of peripheral blood mononuclear cells (PBMC) from 3 ml of whole blood was performed using one of the following methods:

• Red Blood Cell Lysis Buffer (RBCL, BioLegend) was used for lysis of erythrocytes according to the manufacturer’s instructions. Then the cells were washed once with phosphate buffer saline (PBS).

• centrifugation 300 g for 10 minutes. Serum, including interphase, was transferred into PBS and centrifuged 300 g for 10 minutes.

sedimentation method on the density gradient Histopaque- 1077 Hybri-max (Sigma) according to the manufacturer’s instructions.

Cryopreservation of PBMC was performed in a cell freezing medium: 10 % DMSO, 90 % KSR (Thermo Fisher Scientific). Cells were frozen at –80 °C and stored in liquid nitrogen. After thawing, the cells were washed once with PBS.

Cells were counted and resuspended in PBS at a concentration of 1 million cells/ml. Cell fixation, Hi-C library preparation, and data analysis were performed as described in Gridina et al. (2021) using DNase I (Thermo Fisher Scientific) or S1 nuclease (Thermo Fisher Scientific) for chromatin fragmentation. HAPA Hyper prep and QIAseq® FX DNA Library Kit (Qiagen) were used for NGS libraries preparation, according to the manufacturer’s instructions. The DNA concentration was measured using a Qubit 3.0 fluorimeter (Thermo Fisher Scientific). NGS libraries were sequenced on HiSeq XTen (Illumina) with 150 bp paired reads.

## Results and discussion

The first Hi-C step is cells fixation with paraformaldehyde, which is necessary to preserve the native spatial organization of chromatin within the nucleus. Unfortunately, it is not always possible to deliver the sample to the laboratory for fixation on the blood collection day. We decided to systematically estimate the impact of blood storage and preprocessing conditions on the quality of the obtained Hi-C data. Ten conditions were chosen, which included: different methods of PBMC isolation from whole blood; different time and temperature of sample storage; the possibility of freezing PBMC before fixing for long-term storage (see Fig. 1 and the Table).

**Fig. 2.**
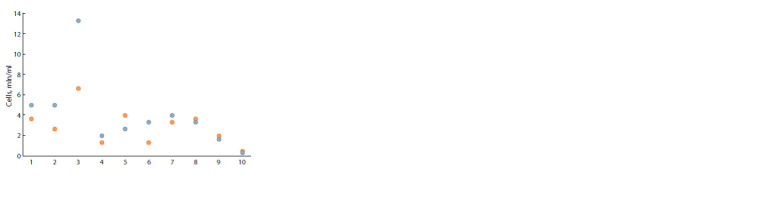
The PBMC count in 1 ml of blood. Colors indicate replicates. The horizontal axis represents the storage and preprocessing conditions described in the Table.

Although blood sampling is a minimally invasive procedure for biomedical diagnostics, it is clear that there are certain limits on the amount of blood that can be obtained from a patient. Especially if the patient is a small child, or has certain problems with the blood coagulation system. Hi-C analysis requires 1.5–2.5 million cells. Normally, 1 ml of blood contains (4–11) × 106 cells. To test each condition, 3 ml of whole blood was taken in two replicates. The PBMC were counted (Fig. 2) after erythrocytes lysis but before cells fixation. A significantly higher number of cells were in the samples processed according to condition #3 (isolation of nuclear elements without RBCL treatment). We did not determine the proportion of living cells during counting. It is possible that dying cells were preserved in samples #3, whereas they were lysed in other cases using RBCL buffer (Brown et al., 2016) or freezing. Supporting the assumption, there were significantly less cells in samples #4 and #10 that were not treated with RBCL but were frozen than in #2.

There were no signs of hemolysis before the start of the isolation of PBMC for all samples except #6, and it was not possible to evaluate this parameter for samples #6. Hemolysis should be avoided, as it is one of the main factors negatively affecting the amount of DNA isolated from blood (Caboux et al., 2012), which may be associated with DNA degradation by nucleases released from degrading cells.

Cell conglomerates were formed in some samples during erythrocyte lysis and subsequent washings. The conglomerates were in both replicates in samples #6, #8, #9 and #10. For these samples, it was not possible to accurately count cells and aliquot them uniformly.

**Fig. 3.**
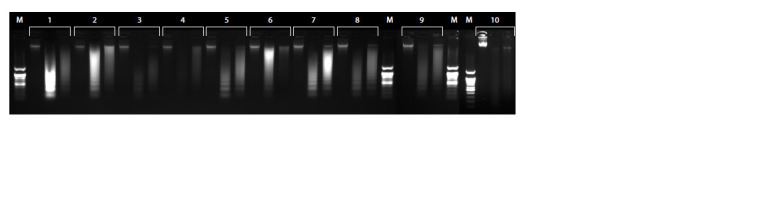
Chromatin fragmentation and ligation controls in Hi-C experiments The numbers represent the storage and preprocessing conditions described in the Table. The order of samples: gDNA, fragmented DNA, ligated DNA. М – DNA ladder 100 bp.

2.5 million fixed cells were taken to prepare Hi-C libraries. To assess the quality of Hi-C libraries (Belaghzal et al., 2017), the following controls were made: genomic DNA, DNA after chromatin fragmentation and after ligation. All controls looked accepted (Fig. 3).

**Fig. 4.**
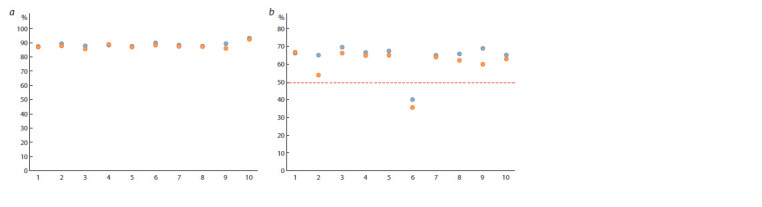
Quality metrics of Hi-C datasets: а, aligned reads; b, cis interactions. Colors indicate replicates. The horizontal axes represent the storage and preprocessing conditions described in the Table.

We sequenced the Hi-C libraries using paired-end reads with a length of 150 bp, mapped the paired-end reads to the human hg19 genome (GRCh_37) and estimated quality metrics of Hi-C datasets. All libraries had a high proportion of aligned reads (Fig. 4, a).

Previously, we have shown (Gridina et al., 2021) that the most important quality metric of Hi-C datasets is the proportion of cis interactions (ratio cis/all (FF and RR orient)) (see Fig. 4, b). It reflects the proportion of Hi-C reads that mapped on the same chromosome among all Hi-C reads. The percentage of cis interactions was comparable for all libraries except samples #6 where it was 40.3 and 35.7 %. It means that these Hi-C data are not informative as most fragments ligated randomly. Blood samples #6 were frozen without a cryoprotectant and stored for 4 days at –20 °C. The observed low percentage of cis interactions might be due to random DNA strand breaks occurring when cells are frozen without cryoprotectants (Narayanan et al., 2001; Peng et al., 2008; Al-Salmani et al., 2011). On the other hand, this method of freezing leads to ice crystals formation inside the cell and, as a result, to the breaking of cellular and nuclear structures (Mazur, 1984). The release of DNA fragments from the nucleus and their ligation in solution can occur in any way, which leads to the formation of non-informative DNA fragments.

## Conclusion

We systematically evaluated various blood samples storage and preprocessing conditions in this work.

As a result, we formulated the following recommendations for the storage and preprocessing of blood samples for Hi-C analysis:

• If it is not possible to deliver the sample on the blood collection day, the samples can be stored at +4 °C for a minimum of 7 days. • It is better to lyse red blood cells with RBCL buffer before cryopreservation. • 1–2 ml of whole blood is sufficient (in a person without signs of leukopenia), but if the sample is going to be stored for more than 48 hours, the volume should be increased up to 4–6 ml. • Never freeze whole blood.

## Conflict of interest

The authors declare no conflict of interest.
